# Elevated CO_2_ concentration affects the defense of tobacco and melon against lepidopteran larvae through the jasmonic acid signaling pathway

**DOI:** 10.1038/s41598-020-60749-1

**Published:** 2020-03-04

**Authors:** Qiang Zhang, Wenting Dai, Xuhui Wang, Jinxin Li

**Affiliations:** 1 College of Life Science and Technology, Xinjiang University, Urumqi, 830046 China; 2 Institute of Agro-products Processing Science and Technolog, Xinjiang Academy of Agricultural and Reclamation Science, Shihezi, 832000 China

**Keywords:** Protein purification, Agricultural genetics

## Abstract

The massive use of fossil fuels since the industrial revolution has led to a rapid increase in the concentration of carbon dioxide (CO_2_) in the atmosphere. What effects elevated CO_2_ concentrations (ECO_2_) have on the defense mechanisms plants employ against insects remains poorly understood. This study showed that ECO_2_ of 750 ± 20 mmol/mol, increased the photosynthetic rate and biomass gain of tobacco and melon plants. However, while mass gain of *Spodoptera litura*, a nocturnal moth in the Noctuidae family, was higher when feeding on tobacco plants under ECO_2_, mass gain of *Diaphania indica* was reduced when feeding on melon plant at ECO_2_ compared to ambient CO2. Plants have many mechanisms to defend themselves against insects. Jasmonic acid (JA) is a crucial element of plant defense against lepidopteran insects. Our study showed that JA levels increased in tobacco plants under ECO_2_ but decreased in melon plants. It is speculated that ECO_2_ changes plant resistance to insects mainly by affecting the JA signaling pathway. Nutrient analysis suggested defensive metabolites rather than changes in the total nitrogen or protein content of the plants led to the changes in plant defense levels under ECO_2_. In summary, ECO_2_ affects the interaction between plants and insects. The results may provide a theoretical basis for studying the changes in crop resistance to pests under ECO_2_ and predicting the impact of ECO_2_ on future agro-ecosystems.

## Introduction

Insect feeding is a major cause of biotic stress to plants. During their co-evolution with insects, plants have developed complex defense systems to resist insect feeding. Almost all plants can be harmed by certain molecules in the oral secretions (OS) of herbivorous insects such as fatty acid-amino acid conjugates (FACs)^1,2^. However, plants can also resist the attack of pests by activating a series of signaling events, including cell membrane depolarization, activation of mitogen-activated protein kinases, and accumulation of stress-related plant hormones^3–5^, which may alter the expression of resistance-related genes, to increase the levels of defensive metabolites such as plants of the Brassicales order^6,7^, agglutinin in tobacco (*Nicotiana glauca*)^8^ and benzoxazinoids in maize (*Zea mays*)^9,10^. Carbon dioxide (CO_2_), a basic substance required for plant photosynthesis, is essential for plant growth and development. The global average atmospheric concentration of CO_2_ hit 409 ppm in February 2017 (Mauna Loa Observatory), which was 47% higher than the 278 ppm in 1750^11^ and is projected to reach 750 ppm by the end of this century^12^. An elevated carbon dioxide (ECO_2_) concentration is expected to have profound effects on many aspects of plant physiology, including increased photosynthetic rate, biomass and seed production^13–15^. For example, previous free-air CO_2_ enrichment (FACE) studies have shown that the yield of staple crops (such as sorghum, cotton, wheat and rice) can be increased by an average of 17% under ECO_2_ (700 ppm)^14^.

ECO_2_ is thought to enhance photosynthetic rate and affect plant-insect interactions of C3 plants^16^. According to the carbon-nitrogen balance theory, increasing C-based metabolites can enhance photosynthesis and thus reduce the relative content of proteins. Since nitrogen (N) is a limiting factor for the growth of many herbivores^17,18^, the compensatory feeding hypothesis suggests that insects may have to consume more foliage to obtain sufficient N-based nutrients (mainly proteins)^19–21^. ECO_2_ associated changes in plant nutrients, especially in protein content, are known to affect plant defense against insects. For example, ECO_2_ reduces the leaf nitrogen of peanut and ramie, resulting in increased food consumption, reduced growth rate and prolonged phlegm time in *Spodoptera litura* and *Achaea janata*^22^.

The phytohormone jasmonic acid (JA) is vital in plant defense against insects. For example, in wild-type tobacco, silencing JA biosynthetic gene lipoxygenase 3 (*LOX*3), or the JA signaling receptor coronatine insensitive 1 (COI1) will reduce plant resistance to *Manduca sexta* larvae, due to decreased defense-related metabolites^23,24^. Similar findings were also reported in tomato (*Solanum lycopersicum*), almond (*Amygdalus communis*), and rice (*Oryza sativa*) *Arabidopsis thaliana* plants harboring mutations in genes that are involved in JA biosynthesis or signaling^25–28^. ECO_2_ can alter plant defense against pests by regulating JA level. For example, ECO_2_ reduces tomato resistance to cotton bollworm by inhibiting JA accumulation^29^. Under ECO_2_ leaf JA and JA-Ile concentrations increase, and may induce the production of flavonoids^30^. ECO_2_ improved the feed intake and reproductive performance of Japanesebeetle (*Popillia japonica Newman*)^31,32^ and western corn rootworm (*Diabrotica virgifeya virgifer*)^33,34^ feeding on soybean (*Glycine max*) in FACE experiments, which is associated with downregulated expression of JA biosynthetic genes lipoxygenase 7 (*LOX*7), *LOX*8, allene oxide synthase (*AOS*) and allene oxide cyclase (*AOC*), and an ethylene (ET) biosynthetic gene 1-aminocyclopropanecarboxylic acid synthase (*ACS*)^34,35^.

There are many other factors that can affect plant and insect growth and their interactions. For example, temperature and climate can affect the growth of plants^36^ and insects^37^, and may also affect the way plants resist insects^38^. ECO_2_ will have an impact on the atmospheric temperature and climate environment, thus indirectly affecting the growth of plants and insects, as well as the resistance of plants to insects. In this study, we selected the more direct factors that affect the growth and interaction of plants and insects to elucidate the mechanism of ECO_2_ affects plants resistance insect through the jasmonic acid signaling pathway.

Tobacco and melon are both important C3 plants. In order to explore how ECO_2_ affects the defense of plants (especially C3 plants) against lepidopteran insects, we studied the interaction of tobacco-*Spodoptera litura* (Lepidoptera: Noctuidae), and melon-*Diaphania indica* (Lepidoptera: Coleoptera) to investigate molecular mechanisms of plant resistance to lepidopteran insects under ECO_2_. The results showed that under an ECO_2_ of 750 ± 20 mmol/mol, the resistance of tobacco to *S. litura* increased, while the resistance of melon to *D. indica* decreased, and the changes in plant resistance and JA level showed the same pattern. More importantly, we demonstrated that ECO_2_ alters plant-to-insect resistance by affecting herbivoryinduced JA level. Based on the correlations of plant total nitrogen, total protein, JA level and the growth of insects, we also found that plants are able to inhibit the growth of *S. litura* and *D. indica* mainly by regulating herbivory-induced JA level, while the changes in leaf total nitrogen or total protein have little effect on the growth of the two insects.

## Results

### Effects of ECO_2_ on photosynthesis of tobacco and melon plants

**Figure 1:**
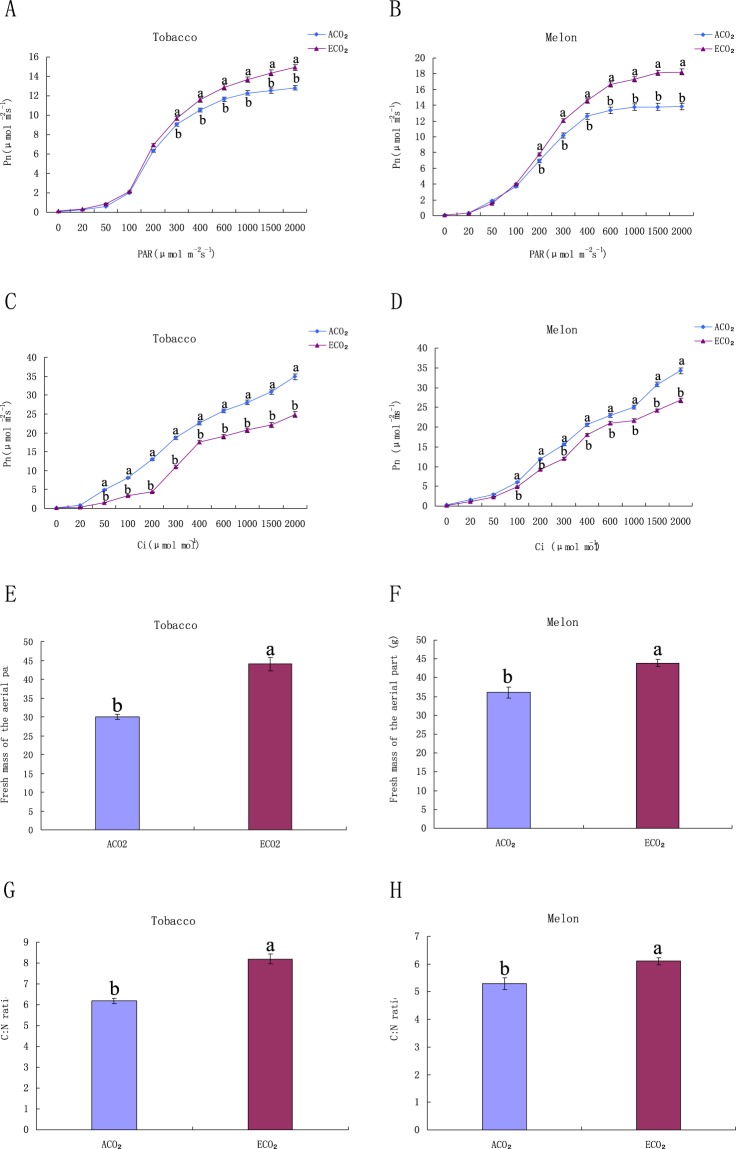
Photosynthetic rates and biomass of tobacco and melon plants under ACO_2_ and ECO_2_. (**A**) Light response curves of five-week-old tobacco plant; (**B**) Light response curves of five-week-old melon plant; (**C**) CO_2_ response curves of five-week-old tobacco plant; (**D**) CO_2_ response curves of five-week-old melon plant; (**E**) Fresh weight of above-ground part of tobacco plant; (**F**) Fresh weight of above-ground part of melon plant; (**G**) C:N ratio of tobacco plant; (**H**) C:N ratio of melon plants. Pn, photosynthetic rate; PAR, photosynthetically active radiation; Ci, intercellular CO_2_ concentration. Diferent letters for each species denote signifcant diferences (*p* ≤ 0.05).

To determine whether ECO_2_ affects the photosynthesis in tobacco and melon plants, the light responsive curve and CO_2_ response curve were plotted. As the light intensity increased, tobacco and melon plants grown under ECO_2_ showed increased photosynthetic rates, compared with the plants grown under ambient CO_2_ (ACO_2_) (Fig. 1A,B). However, according to the CO_2_ response curves, the plants grown under ACO_2_ showed higher photosynthetic rate than the those grown under ECO_2_, with intercellular CO_2_ level increasing (Fig. 1C,D), indicating that ECO_2_ increased the photosynthetic rate but weakened the photosynthetic capacity of tobacco and melon plants, which may be due to the decrease in ribulose 1,5-bisphosphate carboxylase/oxygenase level under ECO_2_^39–41^.

As the photosynthetic rate increased, the fresh weight of tobacco decreased by 49.6% (Fig. 1E), and that of melon increased by 22.3% under ECO_2_ (Fig. 1F). In addition, the C:N ratios of tobacco and melon under ECO_2_ increased by approximately 28.2% and 8.5%, respectively (Fig. 1G,H).

### Resistance of tobacco and melon plants to lepidopteran insects under ACO_2_ and ECO_2_

**Figure 2:**
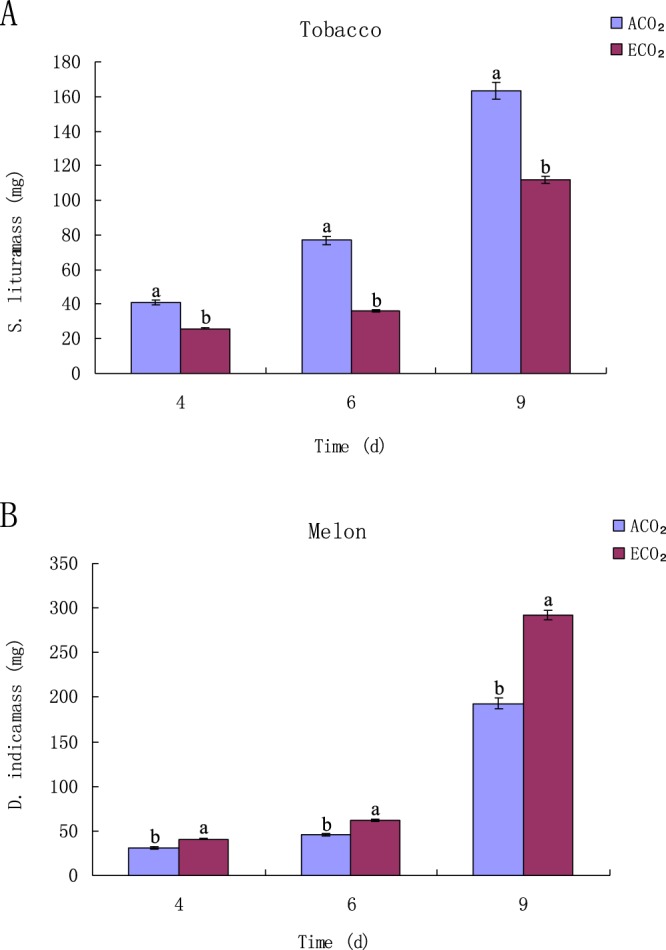
Growth of larvae feeding on tobacco and melon plants under ACO_2_ and ECO_2_. Total mass of *S. litura* on tobacco (**A**) and *D. indica* on melon (**B**) under ACO_2_ and ECO_2_. Diferent letters for each species denote signifcant diferences (*p* ≤ 0.05).

In order to determine whether or not ECO_2_ affects the resistance of tobacco and melon to insects, the mass of insects gained was measured respectively under ECO_2_ and ACO_2_. The results showed that under ECO_2_, the average mass of *S. litura* feeding on tobacco plant decreased by 44%, 46% and 31% on day 4, day 6 and day 9, respectively (Fig. 2A). In contrast, under ECO_2_, the average mass of *D. indica* feeding on melon plant increased by 21%, 27%, and 43% on day 4, day 6 and day 11, respectively (Fig. 2B). The total dry matter of the two insects changed in similar patterns to their mass under ECO_2_ (Fig. 2A,B). These results indicate that ECO_2_ increases the resistance of tobacco to *S. litura* but reduces the resistance of melon to *D. indica*.

### JA level in tobacco and melon under ECO_2_ and ACO_2_

Plant hormones, especially JA, play a crucial role in regulating plant defenses against insects. To uncover the mechanisms by which ECO_2_ affects plant resistance to insects, we determined the levels of JA and JA-isoleucine conjugate (JA-Ile) in tobacco and melon plants treated by different CO_2_ concentrations.

**Figure 3:**
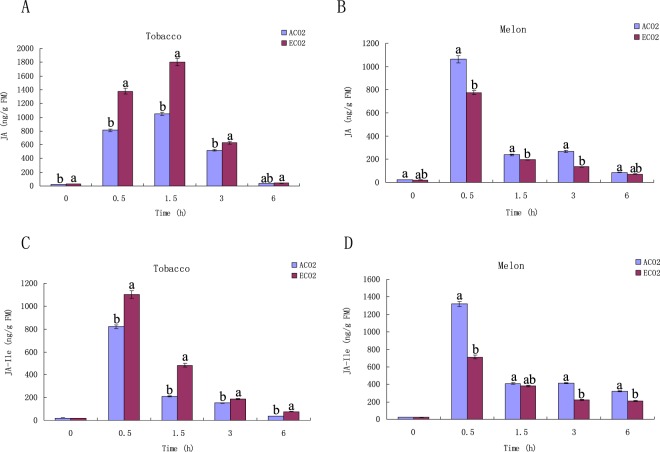
Changes in phytohormones in simulated herbivory treated tobacco and melon plants under ACO_2_ and ECO_2_. Changes in simulated herbivory-induced JA concentration in tobacco (**A**) and melon (**B**); Changes in simulated herbivory-induced JA-Ile concentration in tobacco (**C**) and melon (**D**). Diferent letters for each species denote signifcant diferences (*p* ≤ 0.05).

Since insect feeding is difficult to control, it was simulated by wounding the leaves with a fabric pattern wheel, following which the oral secretion (OS) of *S. litura* or *D. indica* was immediately applied to the wounds. The results showed that the peak value of JA in tobacco plants induced by *S. litura* OS (1 h after induction) under ECO_2_ was 51% higher than that under ACO_2_. However, the peak value of JA in melon plants induced by *D. indica* OS under ECO_2_ was 32% lower than that under ACO_2_ (Fig. 3A,B). JA-Ile conjugate is a JA derivative that binds to the COI1 receptor and thereby activates JA-induced responses^28,42^. We observed that simulated herbivory induced JA-Ile level changed in the same patterns of simulated herbivory induced JA in both plants (Fig. 3C,D).

### Expression of the genes involved in the JA pathway under ECO_2_ and ACO_2_

*LOX* (lipoxygenase)^22,43^, *AOS* (allene oxide synthase)^44^, *AOC* (allene oxide cyclase)^45^, and *JAR* (jasmonic acid resistance)^46,47^ are closely associated to JA and JA-Ile biosynthesis. To assess the effects of ECO_2_ on simulated herbivory -induced JA and JA-Ile levels, the expression levels of these genes in tobacco and melon were determined.

**Figure 4:**
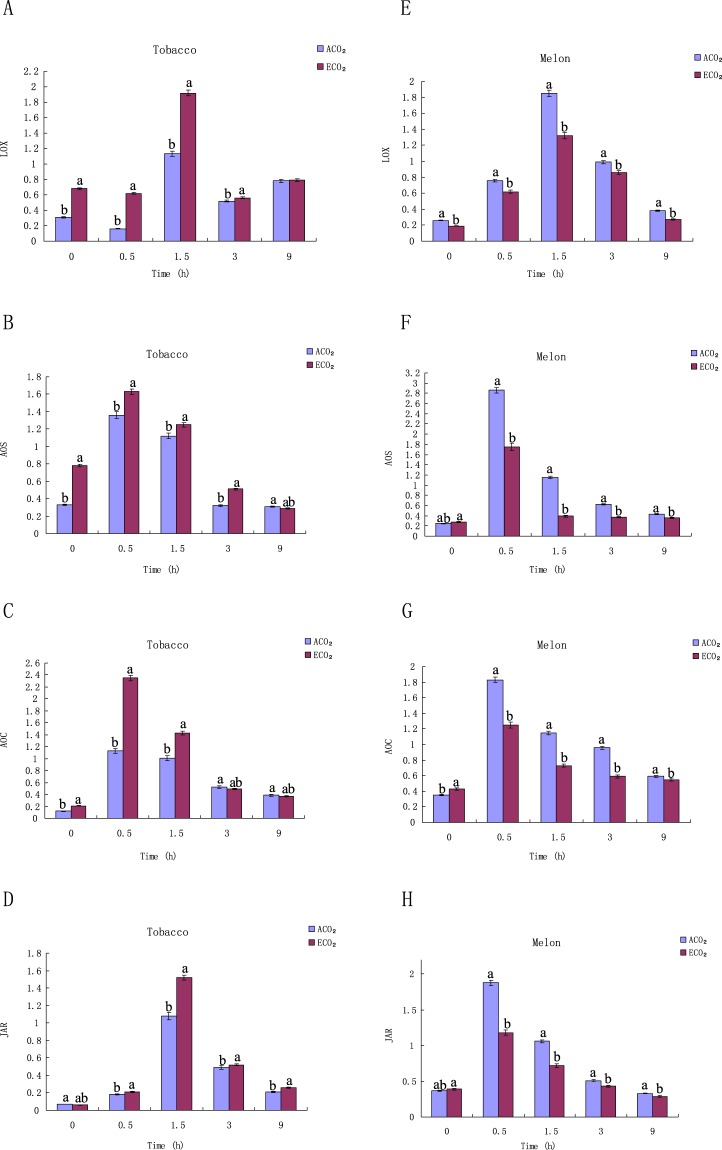
Changes in relative expression levels of defense-related genes in simulated herbivory-treated tobacco and melon plants under ACO_2_ and ECO_2_. Relative expression levels of *NtLOX* (**A**), *NtAOS* (**B**), *NtAOC* (**C**) and *NtJAR* (**D**) in tobacco; Relative expression levels of *CmLOX* (**E**), *CmAOS* (**F**), *CmAOC* (**G**) and *CmJAR* (**H**) in melon. Diferent letters for each species denote signifcant diferences (*p* ≤ 0.05).

In tobacco, the expression levels of Nt*LOX* (1.5 h), Nt*AOC* (0.5 h) and Nt*JAR* (1.5 h) induced by *S. litura* feeding under ECO_2_ were increased by 49%, 88% and 35%, respectively, (Fig. 4A,C,D), compared with those under ACO_2_, while the expression levels of Nt*AOS* changed little (Fig. 4B). In contrast, the peak expression levels of Cm*LOX*, Cm*AOS*, Cm*AOC* and Cm*JAR* in melon induced by *D. indica* feeding decreased by 15%, 35%, 23% and 36% (Fig. 4E–H). These data indicated that herbivory- induced JA in both tobacco and melon changes consistently with the expression levels of JA biosynthesis-involved genes.

### Effects of JA on plant resistance to insects under ECO_2_

**Figure 5:**
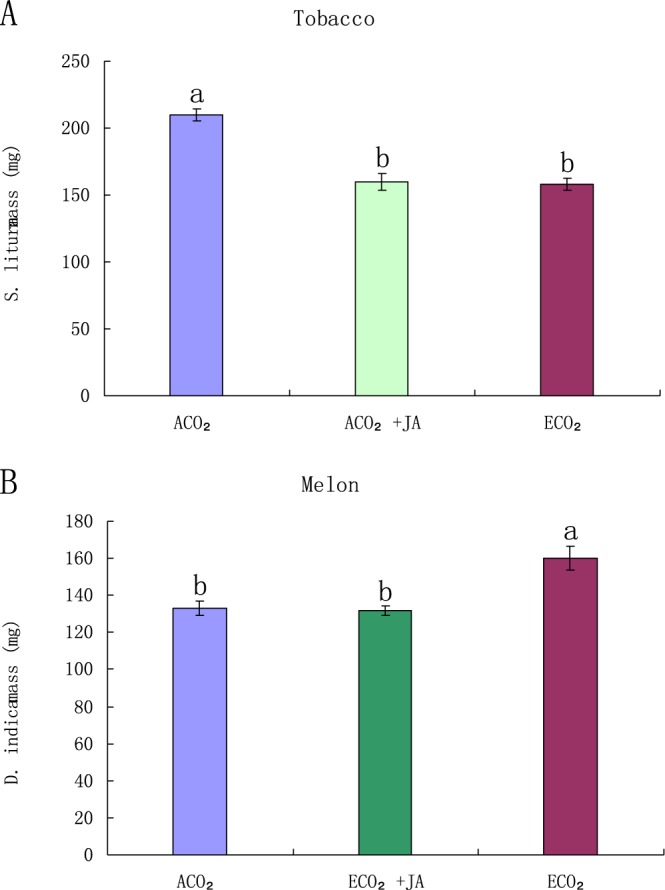
Effect of JA on the growth of *S. litura* feeding on tobacco plants and *D. indica* feeding on melon plants grown under ACO_2_ and ECO_2_, (**A**) Weight of *S. litura* that had fed on untreated tobacco plants, exogenous JA treated tobacco plants under ACO_2_, or untreated tobacco plants under ECO_2_ for 6 days; (**B**) Weight of *D. indica* that had fed on untreated melon plants, exogenous JA treated melon plants under ECO_2_, or untreated melon plants under ACO_2_ for 6 days Diferent letters for each species denote signifcant diferences (*p* ≤ 0.05).

After the JA level in tobacco plants under ACO_2_ was increased using exogenous JA to the same level as in the plants grown under ECO_2_, we found that the mass of *S. litura* feeding on exogenous JA treated tobacco plants was 43% lower than the mass of *S. litura* feeding on untreated tobacco plants under ACO_2_, but showed no significant difference from that under ECO_2_ (Fig. 5A). After the JA level in melon plants grown under ECO_2_ was increased using exogenous JA to the same level as in the plants grown under ACO_2_, we found that the mass of *D. indica* feeding on exogenous JA treated melon plants was 37% lower than the mass of *D. indica* feeding on untreated plants under ECO_2_, but showed no significant difference from that under ACO_2_ (Fig. 5B). The results proved that ECO_2_ changes the resistance of tobacco and melon plants to insects *via* the JA pathway.

### Total nitrogen and protein of tobacco and melon, and the effects on larval growth

**Figure 6:**
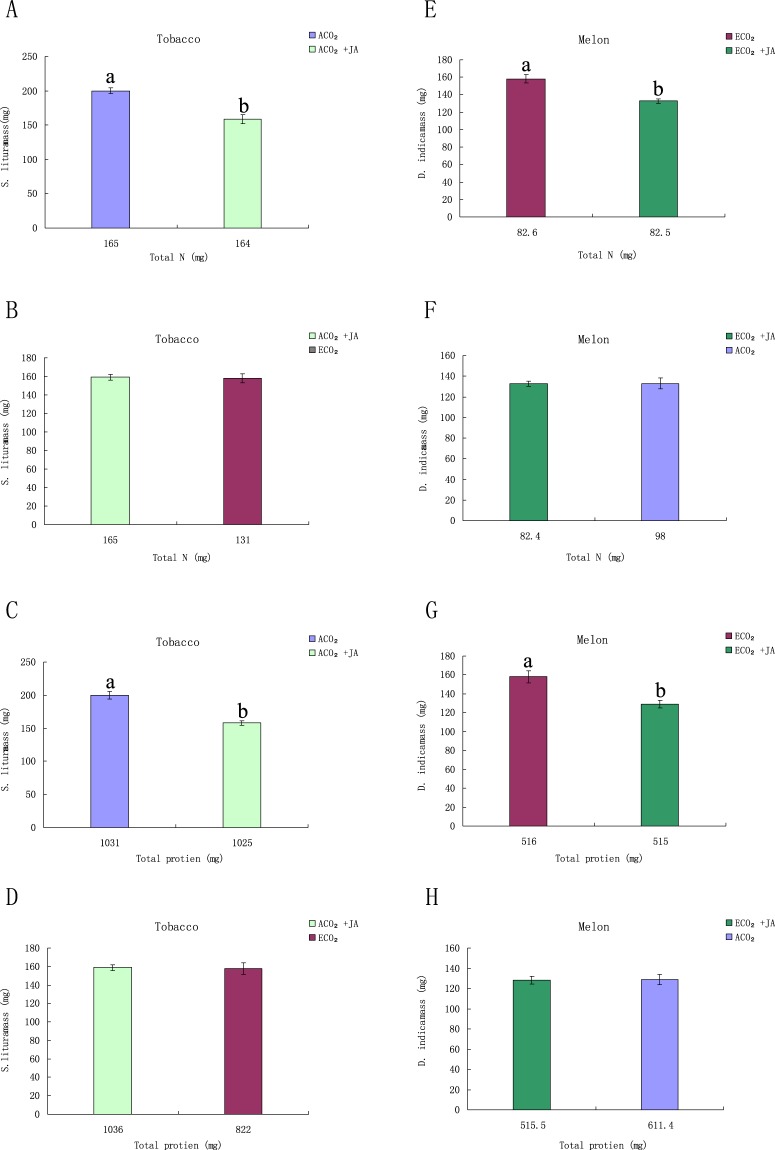
Total nitrogen and protein contents of exogenous JA-treated and untreated tobacco and melon plants under ACO_2_ and ECO_2_, and weight gain of insects in these treatments. (**A**) Total nitrogen of exogenous JA-treated and untreated tobacco plants under ACO_2_, and that of untreated tobacco plants under ECO_2_; (**B**) Total nitrogen and larval biomass of exogenous JA-treated and untreated tobacco plants under ACO_2_ and ECO_2_; (**C**) Total protein of exogenous JA-treated and untreated tobacco plants under ACO_2_, and that of untreated tobacco plants under ECO_2_; (**D**) Total protein and larval biomass of exogenous JA-treated and untreated tobacco plants under ACO_2_ and ECO_2_; (**E**) Total nitrogen of exogenous JA treated and untreated melon plants under ECO_2_, and that of untreated melon plant under ACO_2_; (**F**) Total nitrogen and larval biomass of exogenous JA-treated and untreated melon plants under ACO_2_ and ECO_2_; (**G**) Total protein of exogenous JA-treated and untreated melon plants under ECO_2_, and that of untreated melon plants under ACO_2_; (**H**) Total protein and larval biomass of exogenous JA treated and untreated melon plants under ACO_2_ and ECO_2_; Diferent letters for each species denote signifcant diferences (*p* ≤ 0.05).

Plant nutrients, especially protein, which is the limiting nitrogen source for many herbivores^17^, are critical for larval growth. The total nitrogen and protein contents of both tobacco and melon plants decreased under ECO_2_, which was possibly due to the increased C-based metabolites diluting the contents of proteins and N-based metabolites. Under ACO_2_, the total weight gain of *S. litura* feeding on exogenous JA-treated tobacco plants was 19% lower than that on untreated plants when the larvae ingested the same amount of nitrogen. The total nitrogen content of exogenous JA-treated tobacco plants under ACO_2_ was about 23% higher than that of untreated tobacco plants under ECO_2_, but the *S. litura* larvae that ate the same amount of leaf of the two treatments gained the same weight (Fig. 6A,B), which was similar to the findings in protein content and larval growth (Fig. 6C,D). Similar results were also obtained in melon plants (Fig. 6E–H). These results suggest that it is the JA level rather than leaf nitrogen content or protein content of plants that determines the weight gain of *S. litura* and *D. indica*.

## Discussion

ECO_2_ has a profound impact on plant physiology, especially in C3 plants. It enhanced the defense of tobacco against *S. litura*, but reduced the defense of melon against *D. indica*, suggesting that its effect on plant defense is species-specific. In addition, our study also showed that ECO_2_ alters plant defense against lepidopteran insects mainly by affecting the JA level of plants

ECO_2_ can promote photosynthesis and biomass accumulation of plants. In this study, we found that the photosynthesis rate and growth rate of tobacco and melon plants were both increased under ECO_2_. The study of Zhu *et al*.^48^ showed that the growth rate of soybean seedlings was increased from 6 to 22 μm·min-1 with CO_2_ concentration increasing from 400 to 800 ppm, but began to decrease when CO_2_ concentration exceeded 900 ppm.

The two plant species tomato and melon responded differently to insect feeding under ECO_2_. In detail, the JA content and JA biosynthesis-related gene expression in tobacco plants both increased, while those in melon plants decreased in response to insect OS^29^. It has also been reported that the JA level in tomato plants grown under ECO_2_ reduces in response to cotton bollworm feeding, which suggests that herbivory induced-JA level in plants grown under ECO_2_ changes in a species-specific manner. Our data also showed that the expression of JA and JA-Ile biosynthesis-involved genes in both tobacco and melon under ECO_2_ changed consistently with herbivory induced-JA level, but the mechanism by which ECO_2_ affects plant JA level needs to be further studied. The photosynthetic activity of both tobacco and melon plants also increased significantly under ECO_2_. Considering the key role of chloroplasts in JA biosynthesis and photosynthesis, we speculate that JA level is associated with plant photosynthesis activity. ECO_2_ may change JA level by regulating the content of polyunsaturated fatty acid, which is the substrate for the JA pathway^49^. In addition, environmental CO_2_ concentration may also play an important role in controlling plant defense through a JA-independent pathway. For example, when wounded *Arabidopsis* plants were grown under ECO_2_ they had a lower level of total glucosinolate than those grown under ACO_2_, while they had similar levels of JA and JA-Ile whether grown under ECO_2_ or ACO_2_^50^.

Our results showed that amongst the three factors tested the JA level rather than the content of nitrogen or nutrients is the main factor influencing larval growth. The contents of both nitrogen and proteins in tobacco and melon plants were reduced under ECO_2_, which may be due to the increased C-based metabolites diluting the total nitrogen and proteins in plants. By assessing the effects of plant proteins and defensive metabolites on the defense of exogenous JA treated tobacco and melon plants against pests, we found that defensive metabolites are more influential than protein to plant defense under ECO_2_. Knepp *et al*.^51^ reported that although ECO_2_ had no significant effect on leaf nitrogen of black oak, the larvae feeding on black oak grown under ECO_2_ gained less weight than under ACO_2_. Further analysis revealed ECO_2_ decreased the approximate digestibility of black oak leaf by larvae, leading to a 29% reduction in leaf consumption, a 30% reduction in larval weight gain and a 20% increase in larval mortality under ECO_2_. This suggests the level of defensive metabolites is more influential than the level of proteins in black oak defense mechanisms against pests.

In summary, our data showed that ECO_2_ can affect plant defense against lepidopteran insects, by enhancing the rate of photosynthesis and increasing the plant’s biomass, and altering the level of plant JA induced by insect feeding. In both the crops and insects we studied, JA level played a more important role than leaf protein in determining larval growth under ECO_2_. However, the effect of ECO_2_ on plant defense against insects was shown to be species-specific. Therefore, we believe that it is important to study how the resistance of plants changes in the interaction between important agricultural crops and their major pests, and the changes in crop-pest interaction and in plant resistance to pests under ECO_2_ may provide a theoretical basis for predicting the impact of elevated atmospheric CO_2_ on future agro-ecosystems.

## Materials and Methods

### Plant cultivation

Tobacco ‘NC89’ and melon ‘Jiashi’ plants were grown separately in two climate chambers with the same temperature, light intensity and humidity regimes. Tobacco and melon were planted in each carbon dioxide concentration environment respectively, and the required number of plants were randomly selected during the experiment. The CO_2_ concentration was (400 ± 20 mmol/mol) in one chamber (ACO_2_), and 750 ± 20 mmol/mol in the other (ECO_2_). Tobacco (*Nicotiana tabacum* cv. *Samsun*) plants were grown in 10 L plastic pots. From seed germination, the plants were exposed to a photoperiod 16 hours of light (33 ± 0.05 klux, 28 ± 1 °C) and 8 hours of darkness (20 ± 1 °C) at a relative humidity of 60% ± 2%, watered once every 6 days and fertilized with 1 g/L nitrogen, phosphorus, potassium and trace elements at a ratio of 20:20:20:0.5, once every two weeks. The seeds of Jiashi melon were seeded in a 128-well plate containing a mixed matrix (meteorite: perlite = 1:1), After germination, 1/2 of the nutrient solution (N:P:K = 20:20:20) was watered every 2 days, when the seedlings grew to two leaves, they were transplanted into 20 L PVC barrels (1 plant per barrel), other cultivation environment conditions are the same as tobacco.

### Determination of photosynthetic parameters

Photosynthetic rate was measured using a LI-COR 6400 portable photosynthesis system (LI-COR Biosciences). The photosynthetic parameters were determined using the fourth true leaf of tobacco plants and the third and fourth true leaves of melon plants. The CO_2_ concentration in the chambers was set to 400 mmol/mol, and the Pn (photosynthetic rate) and PAR (photosynthetically active radiation) values at 11 light intensities (0, 20, 50, 100, 200, 300, 400, 600, 1000, 1500 and 2000 mmol·m^−2^·s^−1^) were measured to plot the light response curves. To fit CO_2_ response curves, the Pn and Ci (intercellular CO_2_ concentration) were measured at 12 different CO_2_ concentrations (0, 50, 100, 200, 300, 400, 600, 800, 1000, 1200, 1600, 2000 mmol/mol), and at a light intensity of 1000 mmol·m^−2^·s^−1^. During the measurements, the chamber temperature was controlled at 27 °C and relative humidity at 60%.

### Larva feeding and simulated insect feeding

*S. litura* and *D. indica* eggs were purchased from Genralpest Biotech (http://genralpest.b2b.hc360.com/). Following emergence the larvae were reared on artificial diets for two days and then transferred onto five-week-old plants. To determine the effect of ECO_2_ on plant resistance to insects, 15 tobacco plants were infested. with 50 to 80 *S. litura* larvae (3 to 4 larvae per plant), and 15 melon plants were infested. with 50 to 80 *D. indica* larvae (3 to 4 larvae per plant). And their weights were measured on the indicated days.

The oral secretions (OS) of 4^th^- and 5^th^-instar *S. litura* and *D. indica* larvae were collected from the tobacco and melon plants respectively, and stored at −80 °C. To simulate insect feeding, the leaves were wounded with a fabric pattern wheel, before 20 μL of *S. litura* or *D. indica* OS was gently rubbed onto the freshly created wounds, and these experiments repeated 3 times.

### Plant hormone analysis

Approximately 100 mg of leaf tissue of each sample was ground in liquid nitrogen, before 1 mL of ethyl acetate containing 20 ng of internal standards D4-SA, D5-JA and JA-13C6-Ile was added, thoroughly mixed, and centrifuged at 13 000 g for 10 minutes at 4 °C. The supernatant was transferred to a new tube and evaporated to dryness using a vacuum concentrator (Eppendorf, Hamburg, Germany) at 30 °C. Leaf samples were immersed in 0.6 mL of methanol- water mixture (70:30, *v/v*) and centrifuged at 13 000 g for 10 minutes at 4 °C. The supernatant was transferred to a glass vial and injected into the Ultra Performance Liquid Chromatography-Mass Spectrometry/MS system (LCMS-8040 system, Shimadzu). The peak areas of the internal standard and each standard compound were used to calculate phytohormone concentration.

### Protein extraction and quantification of total C and N

Approximately 50 mg of leaf sample that had been ground was mixed with 0.3 mL of extraction buffer containing 0.1 M Tris-HCl (pH 7.6), 5% (*m/v*) polyvinylpolypyrrolidone, 2 mg/mL phenylthiourea, 5 mg/mL diethyldithiocarbamate, and 0.05 M Na_2_EDTA, and then immediately centrifuged at 16 100 g for 20 minutes at 4 °C. After that, 200 mL of the supernatant was transferred to a new tube. Protein concentration was determined photometrically (595 nm) in a 96-well plate using Bradford 1x Dye Reagent (BIO-RAD).

For the measurement of total C and total N, leaves samples were dried in an oven at 100 °C for 2 days, and pulverized, before total C and N contents were quantified using an Elemental Combustion System (Elementar, vario MICRO).

### RNA extraction and Real-time quantitative RT-PCR expression analysis

Total RNA samples were extracted from leaf material with the EasyPure Plant RNA Kit (TRANS, China) following manufacturer protocol. To amplify the selected genes, cDNA was amplified by PCR using the following primerswere listed in (Table 1) and were synthesized with the EasyScript First-Strand cDNA Synthesis SuperMix (TRANS China), to be used as the template for RT-PCR^52^. Real-time quantification RT-PCR reactions were performed in Bio-RAD MyiQTM Real-time PCR Detection System (Bio-Rad, USA) using the TransStart Top Green qPCR SuperMix (TRANS, China) according to the manufacturer’s instructions and the tobacco *elongation factor 2* and melon *acti*n (CmActin) was employed as the internal control for RT-PCR analysis^53^. Amplifcation was carried out through initial denaturation at 94 °C for 2 min, followed by 38 cycles of denaturation at 94 °C for 30 s, annealing at 57 °C for 30 s, and elongation at 72 °C for 2 min. The PCR products from each amplifcation reaction were separated on 2.5% (w/v) agarose gels.

**Table 1:** Primers used in this study.

Gene name Gene accession No Primer sequence
NtLOX NM_001325784.1 F5′-ATCGCCCTACATTAAGCCGA-3′
R5′-GCTTCTTTCCAAACCTCGCA-3′
NtAOS NM_001325375.1 F5′-GCCAAACGCGACCTTATGAT-3′
R5′-CCACAAAATCCTTTCCGGCA-3′
NtAOC NM_001324978.1 F5′-GAGCCAGCACCTGAAGCTAA -3′
R5′-TTCTCCGGAAATGACCCCAC-3′
NtJAR DQ359729.1 F5′-GCCTCCCGAGCTTGTTACAT-3′
R5′-GACCGTCTAAATTTTCCATGAGA-3′
NtEF2 XM_016589436.1 F5′-TGCTGGTACACAAGCTCATCAA-3′
R5′-AGTCACTGCCTGCTTCAAACC-3′
CmLOX MELO3C014630 F5′-TGATGCTACCCAAGCGATGT -3′
R5′-ATGGTGGACTGAGATTAGAACG-3′
CmASO MELO3C018833 F5′- CCCCAGCAATCGAATCAT-3′
R5′-CCCGAGCGAGAAGAACAA-3
CmAOC MELO3C003015 F5′-CTGCTGCAACTTGACCTG-3′
R5′-GCTTCTACTTCGGCGATT-3′
CmJAR MELO3C010083 F5′- TATTATACAACGAGCATC -3′
R5′-AAAGTGTCTACAGGAAAT-3′
CmActin MELO3C011913 F5′- CCAAAGGCTGCAAGAATAGC-3′
R5′-TTTGACCTTTGGGTGGGTAG-3′
